# *Chickpea chlorotic dwarf virus*: An Emerging Monopartite Dicot Infecting Mastrevirus

**DOI:** 10.3390/v11010005

**Published:** 2018-12-21

**Authors:** Surapathrudu Kanakala, Paul Kuria

**Affiliations:** 1 Department of Plant Pathology and Microbiology, Iowa State University, Ames, IA 50010, USA; 2 Kenya Agricultural and Livestock Research Organization, Nairobi 00200, Kenya; kuriapk@gmail.com

**Keywords:** *Mastrevirus*, *Betasatellite*, *Chickpea chlorotic dwarf virus*, CpCDV, genetic diversity, infectivity, recombination, RNA interference, resistance

## Abstract

Chickpea stunt disease (CSD), caused by *Chickpea chlorotic dwarf virus* (CpCDV) is a threat to chickpea production leading to yield losses of 75–95%. *Chickpea chlorotic dwarf virus* is a monopartite, single-stranded circular DNA virus in the genus *Mastrevirus* and family *Geminiviridae*. It is transmitted by *Orosius albicinctus* in a circulative (persistent) and nonpropagative manner. Symptoms of CSD include very small leaves, intense discoloration (yellowing (*kabuli* type) and reddening (*desi* type)), and bushy stunted appearance of the plant. Presently, CpCDVs occurs in Africa, Asia, Australia, and the Middle East, causing extensive losses on economically important crops in in the families Fabaceae, Asteraceae, Amaranthaceae, Brassicaceae, Cucurbitaceae, Caricaceae, Chenopodiaceae, Leguminosae, Malvaceae, Pedaliaceae, and Solanaceae. High frequency of recombinations has played a significant role in the wide host range, diversification, and rapid evolution of CpCDVs. This review highlights the extensive research on the CpCDV genome diversity, host range, plant–virus–insect interactions, and RNA interference-based resistance of CpCDV, providing new insights into the host adaptation and virus evolution.

## 1. Introduction

Chickpea (*Cicer arietinum* L.) is an important pulse crop grown and consumed all over the world. As the world’s population rises, the demand for grain legumes is also rising, and it is a permanent challenge to meet increasing demands. However, abiotic and biotic factors affect plant growth and pose a threat to sustainable agriculture and food production. Pathogens include fungi, bacteria, viruses, nematodes, and mycoplasma [1,2]. Several insect-transmitted viruses have been known to cause diseases in chickpea under field conditions: aphid-transmitted (virus in the families *Bromoviridae*, *Luteoviridae*, *Nanoviridae*, and *Potyviridae*) and leafhopper-transmitted (virus in the family *Geminiviridae*) viruses can lead to significant economic loss [3]. Among the leafhopper-transmitted viruses reported in chickpea, the most important and threatening viral disease is chickpea stunt disease (CSD).

CSD was recognized as a serious endemic problem in India as early as the 1970s [4]. The viruses, pea leaf roll virus in Iran [5]; subterranean clover red leaf virus (SCRLV), a strain of soybean dwarf virus, and beet western yellows virus (BWYV) in California [6,7]; as well as BWYV and bean leaf roll virus (BLRV) in Spain [8] were found to be associated with the chickpea stunt disease and discoloration symptoms. In India, BLRV was thought to be associated with the disease until 1993. CSD was first identified in India, and later the virus causing disease was identified as CpCDV and was shown to be transmitted in a persistent manner by the leafhopper *O. albicinctus* [9]. A survey showed CSD to be prevalent in the Indian states of Andhra Pradesh, Gujarat, Haryana, Madhya Pradesh, and Punjab, causing 75–95% losses in yield [10,11,12]. Later, Nahid et al. (2008) [13] in Pakistan and Kanakala et al. (2012) [14] in India characterized the CpCDV, which was identified as one of the etiological agents of stunt disease belonging to the genus *Mastrevirus* of the family *Geminiviridae*.

The family *Geminiviridae* comprises monopartite or bipartite circular single-stranded circular DNA (ssDNA) viruses characterized by their 22 × 38 nm^2^ germinate particles comprised of two joined incomplete icosahedra (T = 1) encapsidating an ssDNA genome molecule of about 2.8 kb [15]. The family is further divided into nine genera (i.e., *Becurtovirus*, *Begomovirus*, *Capulavirus*, *Curtovirus*, *Eragrovirus*, *Grablovirus*, *Mastrevirus*, *Topocuvirus*, and *Turncurtovirus*) on the basis of host range, insect vector, genome structure, organization, and genome-wide pairwise sequence identities [16]. The genomes of the genus *Mastrevirus* consists of a single component (monopartite) of circular ssDNA, of 2.5–2.7 kb length. Mastreviruses are transmitted by leafhoppers in a circulative (persistent) and nonpropagative manner [16].

*Mastrevirus* is the second-largest genus in the family *Geminiviridae*, with 37 species [16] known to infect either monocotyledonous or dicotyledonous plants in association with ssDNA satellite molecules [17,18], of approximately half the size of their helper virus genome. As dicot-infecting mastreviruses are important pathogens in agriculture, this review will mainly focus on the new discoveries, diversity of CpCDVs, geographical distribution, host range, interaction with satellite molecules, and role of recombination in CpCDV complex evolutions and new strategies for their management.

## 2. Disease Symptoms

**Figure 1:**
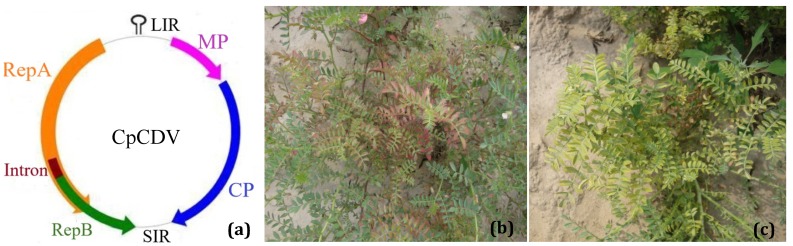
(**a**) Schematic diagram of the CpCDV genome representing virion and complementary open reading frames, Movement protein (MP), Capsid protein (CP), and Replication associated protein A and B (RepA and RepB), LIR and SIR—large and small intergenic regions. Intron and stemloop region are indicated. Chickpea plants showing (**b**) leaf reddening and (**c**) leaf smalling; stunting and proliferation of axillary shoot.

The symptoms of the disease caused by the dicot-infecting mastreviruses are yellowing, stunting, and dwarf symptoms in tobacco when infected by *Tobacco yellow dwarf virus* (TYDV) [19]. *Bean yellow dwarf virus* (BeYDV, now CpCDV-B [20]) in French bean causes stunting, chlorosis, and leaf curling symptoms [21]. The characteristic CSD symptoms are extreme stunting, shortening of internodes, reduction of leaf lamina, bushy and brittle appearance of plants, phloem browning in the collar region, leaf reddening in the case of indigenous types (*desi*), and yellowing in introduced (*kabuli*) types [9]. Field chickpea plants were found with symptoms like chlorosis, leaf smalling, and reddening of the chickpea leaves in Pakistan [13] and India [14] (Figure 1b,c). The yield loss is nearly total if the infection occurs in the early stage of growth; if infection occurs at the flowering stage, the yield loss is 75–90% [10].

In 2013, hot pepper plants growing in India showed stunting and upward leaf curling [22]. CpCDV-A infected different squash plants (*Cucurbita pepo* L.) with severe infections showing leaf curling, yellow mottling, stunting, and reduced fruit set were observed in Egypt [23]. Watermelon fruits showing the symptoms hardness and discoloration of the flesh, whitish inserts, and deformation of fruits and seeds was observed in the Tunisia area [24]. Similarly, CpCDV-C infected field spinach plants showed typical symptoms including leaf curling, vein thickening, and yellowing [18]. More recently, tomato and papaya plants infected with CpCDV showed severe symptoms of leaf dwarfing, curling, and yellowing in Central and Eastern Burkina Faso [25].

## 3. Genome Organization and Protein Functions

### 3.1. Genome Organization

The genome of CpCDV consists of a single component (monopartite) circular ssDNA, 2.5–2.7 kb. There are four open reading frames (ORFs), two on the virion sense strand (ORF V1, capsid protein; ORF V2, movement protein) and two ORFs in the complementary sense strand (ORF C1: C2), from which the replication initiation protein is expressed by transcript splicing (Figure 1a) [26]. The genome codes for movement (ORF V2 nt co-ordinates 133–411, 10 kDa) and capsid protein (ORF V1 nt co-ordinates 424–1161, 26.6 kDa) on the viral strand and replication initiation protein (ORF C1/C2, nt co-ordinates to 2324–1320, 36.3 kDa) on the complementary strand. The large intergenic region (LIR) of ~308 nt contains a characteristic stem loop structure with the invariant nonanucleotide sequences (TAATATT↓AC). However, a rare nonanucleotide TAATGTTAC was uncovered in LIR of CpCDV isolated from watermelon in Tunisia [24]. The LIR also contains the TATA boxes and CA motifs. The putative Rep-binding site, “TGGAGGCA” is present as a tandem repeat 106 nt upstream of the nonanucleotide loop. There is a small intergenic region (SIR) at the 3′ end of V1 and C2 ORFs. A short complementary sense DNA primer containing 5′ ribonucleotides is found encapsidated along with the genomic DNA. This primer is complementary to sequences in the SIR region of the genome [27].

### 3.2. The Rep Protein Complex

The Rep protein, which is the only protein responsible for the initiation of replication, is a ~39 kDa protein comprising ~334 amino acids. In silico analysis of the Rep protein (CpCDV-A, -B, -C, -D, -E, -F, -G, -H, -I, -J, -K, -L, -M, -N, -O, -P, -Q, -R, and -S strains) revealed the presence of motif I (FLTYP), motif II (HY/CHALI/VQ or HYHASYS) and motif III (VLD/EYIS) [28,29]. The analysis also revealed the domain LRCHE at the N-terminal region, which is involved in binding with retinoblastoma protein (RBR). The NTP-binding sites (Walker A (GPT/NRTGKT or DQL/VVPERQ/P) and Walker B (NV/IIDDI) [30] motifs which are common to the Rep protein of geminiviruses were also located in CpCDV. The N-terminal region of the Rep protein is predicted to bind the iteron sequences in the intergenic region. This region is referred to as the iteron-related domain (IRD) [31] and is present in the N-terminal region which was identified to be the amino acid residues FRF/LQ in CpCDV.

### 3.3. The Capsid Protein (V1) and Movement Protein (V2)

The capsid protein (V1) of *Mastrevirus*, like other geminiviruses, is a multifunctional protein. It is a structural protein and is required for encapsidation, accumulation of ssDNA, vector-mediated transmission, nuclear import of genomic DNA, and cell-to-cell movement of the viral genome. The movement protein encoded by the virion sense strand ORF V2 is positioned upstream of the capsid protein gene [32].

## 4. Genetic Diversity, Host Range, and Evolution

### 4.1. Variants of CpCDV

**Figure 2:**
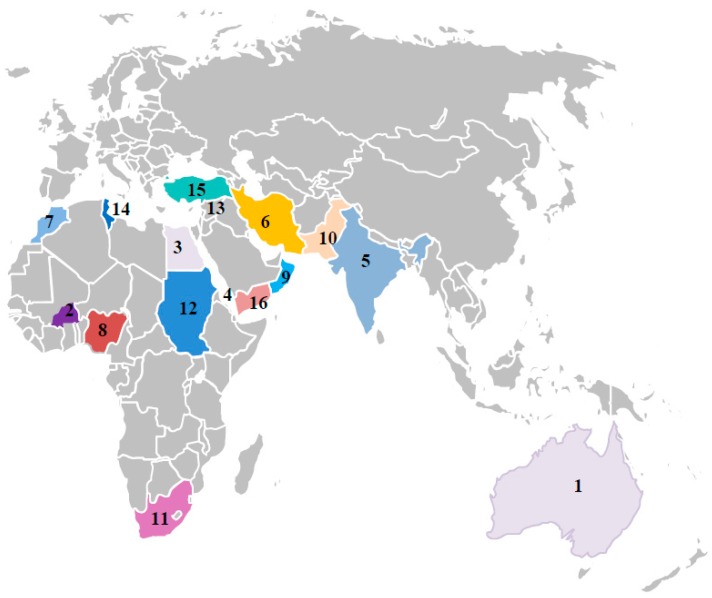
Geographical distribution of dicot infecting mastreviruses across the old world. 1. Australia- CpCAV, CpCV-A,B,C,E,F, CpRLV, CpYV and TYDV; 2. Burkina Faso- CpCDV-Q,R; 3. Egypt- CpCDV-A, 4. Eritrea- CpCDV-C,D,E,F,H,I,K,M,N,O,P; 5. India-CpCDV-C,D; 6. Iran- CpCDV-A; 7. Morocco- CpCDV-D; 8. Nigeria- CpCDV-S; 9. Oman- CpCDV-F; 10. Pakistan- CpCDV-B,C,D,F,H,I, CpYDV; 11. South Africa- CpCDV-B; 12. Sudan- CpCDV-C,D,E,F,H,I,K,M,N,O,P; 13. Syria- CpCDV-A,F; 14. Tunisia- CpCDV-A,H; and 15. Turkey- CpCDV-A; 16. Yemen- CpCDV-F.

A total seven dicot-infecting *Mastrevirus* species have been reported from Australia, the Middle East, and the Indian subcontinent. Among them, six viruses (*Chickpea chlorosis virus* (CpCV), *Chickpea chlorosis Australia virus* (CpCAV), *Chickpea redleaf virus* (CpRLV), *Chickpea yellows virus* (CpYV), *Chickpea chlorotic dwarf virus* (CpCDV), and *Chickpea yellow dwarf virus* (CpYDV)) were characterized from chickpea and TYDV from tobacco [16]. Recently, Muhire et al. (2013) [20] reclassified these viruses causing chickpea stunt in Africa and Asia, on the basis of 78% nucleotide identity in the genomic DNA and grouped all the South Asian mastreviruses as “*Chickpea chlorotic dwarf virus*”. The species demarcation criteria of mastreviruses (www.ictvonline.org) are based on their nucleotide sequence identity, *trans*-replication of genomic components, capsid protein characteristics, transmitting vector species, natural host range, and symptom phenotype. To date, 19 strains of CpCDV (CpCDV A to S strains) have been reported in this genus. Until 1994, presence of CpCDV was limited to the Indian subcontinent, but they were later found in the Middle East and Africa. The emergence of new dicot-infecting mastreviruses have been variously reported in recent years (2013–2017) throughout South and North Africa, South Asia, and the Arabian Peninsula (Figure 2) [13,14,23,24,25,33,34,35,36,37].

### 4.2. Host Range

Until the last decade, the majority of the mastreviruses were known to infect monocots, with only a few members infecting dicots. TYDV (syn. bean summer death virus) causes diseases in green bean (*Phaseolus vulgaris*) and tobacco (*Nicotiana tabacum*) in Australia [38,39,40,41]. Liu et al. (1997) [21] and Rybicki and Pietersen (1999) [42] reported a South African geminivirus, BeYDV infecting French beans showing stunting, chlorosis, and leaf curl symptoms.

BeYDV/CpCDV-B systemically infects *N. benthamiana, N. tabacum, Lycopersicon esculentum, Datura stramonium*, and *Arabidopsis thaliana* [21]. In Australia, along with chickpea, hosts like faba bean (*Vicia faba*), canola (*Brassica napus*), mustard (*B. juncea*), coriander (*Coriandrum sativum*), spotted medic (*Medicago Arabica*), subterranean clover (*Trifolium subterraneum*), and turnip weed (*Rapistrum rugosum)* were identified as natural hosts for *Chickpea chlorosis virus* [43], and three distinct *Mastrevirus* species are known to infect dicotyledonous hosts such as chickpea, bean, and tobacco [44,45]. In the past decade, the importance and diversity of mastreviruses infecting various crops have increased. In addition to chickpea, CpCDV sporadically affects sugar beet (*Beta vulgaris*) [46], *P. vulgaris* (bean), *V. faba* (fava bean), *Pisum sativum* (field pea), *Lens culinaris* (lentil) [35], cotton [47], pepper [22,48], and the weeds *Sesbania bispinosa* [13] and *Xanthium strumarium* [49].

**Figure 3:**
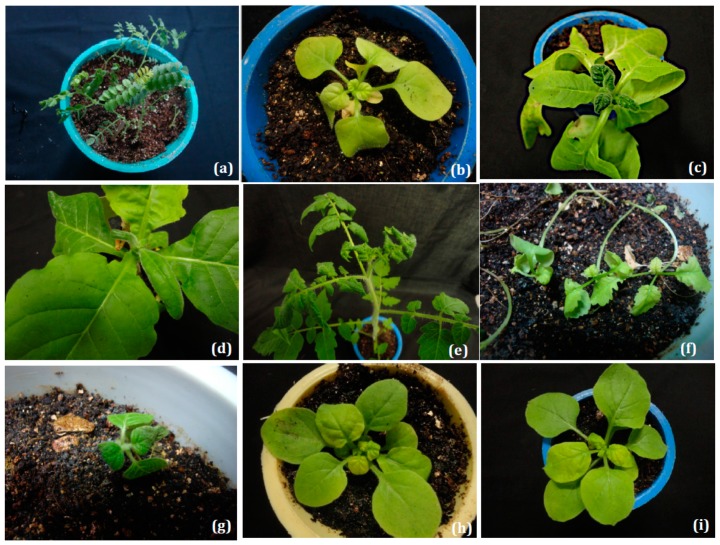
Symptoms in various hosts with agro-inoculated CpCDV-C. (**a**) Chickpea plants showing extreme reduction in leaf size, stunting, yellowing of terminal leaves, dwarfing and proliferation of axillary buds, drying, and eventual death. (**b**) *N. benthamiana* showing severe stunting, chlorosis, downward folding of margin and reduction of leaf lamina, (**c**) *N. glutinosa* showing severe stunting, small thick green leaves and backward curling of apical leaves followed by reduction in shoot elongation; (**d**) *N. tabacum* showing thickening of leaves, crumpling, and reduction in leaf lamina. (**e**) Tomato plants showing young leaves became thick, dark green and mild backward leaf curling (**f**) mustard (Family *Brassicaceae*) plants showed typical chlorosis, downward marginal folding, and stunted (**g**) Sesame (Family *Pedaliaceae*, variety Uma), produced very severe symptoms with thickening of leaves, downward folding, crumpling, and reduction of leaf lamina. *N. benthamiana* agro-inoculated with CpCDV + ToLCNDV DNA B (**h**) and CpCDV+CLCuMuB-[IN:Sr:02] (**i**) showing typical CpCDV symptoms.

CpCDV has been rapidly extending their host range and spreading to new geographical regions. In the past six years, reports from the Middle East and Africa have identified CpCDV infection in various agriculturally important crops. Moreover, further spread has occurred, with recent field reports of CpCDV in Egypt infecting squash [23]; watermelon in Tunisia [24]; cotton [47], tomato, okra [50,51], cucumber [52], and spinach [18] in Pakistan; pepper in Oman [48]; *Carica papaya* in Nigeria and Burkina Faso [25,33]; *V. faba* [35] and wild legumes (*Acacia* spp. *Cajanus cajan*, *Dolichos lablab*, *Rhynchosia minima*) in Sudan [53]; and *Lens culinaris* in Sudan and Pakistan [35] (Table 1). Focusing on individual CpCDV strains, except CpCDV-L, Q, R, and S strains, all strains were characterized from chickpea (Table 1). More recently, CpCDV-A was characterized from *Citrullus lanatus* and squash [23,24]. CpCDV-C has seven new hosts (cucumber, cotton*,* okra, pepper, beans, lentils, and tomato). The process of introducing the viral genome into plants mediated by the Ti plasmid of *Agrobacterium* is termed as “agroinfection”, which was first demonstrated with maize streak virus (MSV) [54]. Two hosts—mustard (Family *Brassicaceae*) and sesame (Family *Pedaliaceae*, variety Uma)—were found to be agroinfectable with CpCDV-C (Figure 3f,g). It has been suggested that CpCDV has a broader host range than other dicot-infecting *Mastrevirus* species. Overall, CpCDV were identified in eleven different subfamilies (*Asteraceae*, *Amaranthaceae, Brassicaceae, Cucurbitaceae, Caricaceae, Chenopodiacea*e, *Fabaceae, Leguminosae, Malavaceae, Pedaliaceae*, and *Solanaceae*).

Horn et al. (1993) [11] reported that leafhopper *O. orientalis* (later names as *O. albicinctus*) successfully transmitted the CpCDV to a wide range of hosts belonging to the families *Solanaceae*, *Leguminosae*, and *Chenopodiaceae,* and they found that the virus was efficiently transmitted with a median acquisition access period (AAP), inoculation access period (IAP), and latency period (LP) of 8, 2.3, and 27.7 h, respectively. However, leafhopper transmission assays have not been conducted for all the hosts listed in Table 1 above. Therefore, more studies will reveal the efficacy of leafhoppers in transmitting CpCDV across multiple hosts.

#### Infectivity of Cloned Components

The family *Geminiviridae* consists of viruses which are transmitted by the vector, and most of them are not sap-transmitted, as the viruses are confined to phloem parenchymatous cells. In these cases, rubbing of the leaves with DNA does not work, as the viral DNA needs to reach the phloem tissue for its survival. This problem of virus delivery has been circumvented by *Agrobacterium*-mediated delivery of the viral genome.

*N. benthamiana* infected with clones CpCDV-A showed typical symptoms of yellowing, stunting, and crumpling of newly emerging leaves [24,55]. With CpCDV-B inoculation on *N. benthamiana*, *N. tabacum*, *L. esculentum*, *D. stramonium*, and *A. thaliana* plants became stunted, leaves developed interveinal chlorosis, and they exhibited severe downward curling symptoms [21]. Inoculation of CpCDV-C on *N. benthamiana* resulted in intense yellowing and downward leaf curling (Figure 3b) [14]*. N. glutinosa* showed severe stunting, small thick green leaves, and backward curling of apical leaves followed by a reduction in shoot elongation (Figure 3c) [14], and *N. tabacum* resulted in reduced apical leaves, dark green color, and downward leaf curling (Figure 3d) [14]. Young unfurling leaves became thick, dark green, and had mild backward leaf curling in CpCDV-C agroinoculated tomato plants (Figure 3e) [14]. Chickpea plants showed foliar yellowing and reduced leaf size, and plants were stunted [13].

In 2013, Kanakala et al. (2013) [56] observed differences in symptom phenotype when the viral genome was delivered through *Agrobacterium* in comparison with field infection. Kanakala et al. (2013) [56] showed the proliferation of axillary shoots with very small leaves, intense discoloration, and bushy stunted appearance of the plant as characteristic symptoms in both *kabuli* and *desi* genotypes tested. The reddening symptom seen in *desi* type in field conditions was not seen in agroinoculation. Interestingly, highly susceptible genotypes screened in this study dried after 25 days post inoculation (dpi). The death of virus infected chickpea plants was not observed under field conditions. The drying and death in agroinoculated plants might be due to the high concentration of viral inoculum introduced through direct *Agrobacterium* inoculations. CpCDV-C agroinoculated mustard (Family Brassicaceae) plants showed typical chlorosis, downward marginal folding, and were stunted (Figure 3f). Agroinoculated CpCDV-C in sesame (Family Pedaliaceae, variety Uma) produced very severe symptoms with thickening of leaves, downward folding, crumpling, and reduction of leaf lamina (Figure 3g). Similarly, CpCDV-A-agroinoculated watermelons showed yellowish/whitish areas or stripes in the flesh, which was discolored (i.e., orange instead of red) and, in some cases, displayed a clearly deformed shape [24].

### 4.3. Phylogenetic Relationships and Detection of Recombination

**Figure 4:**
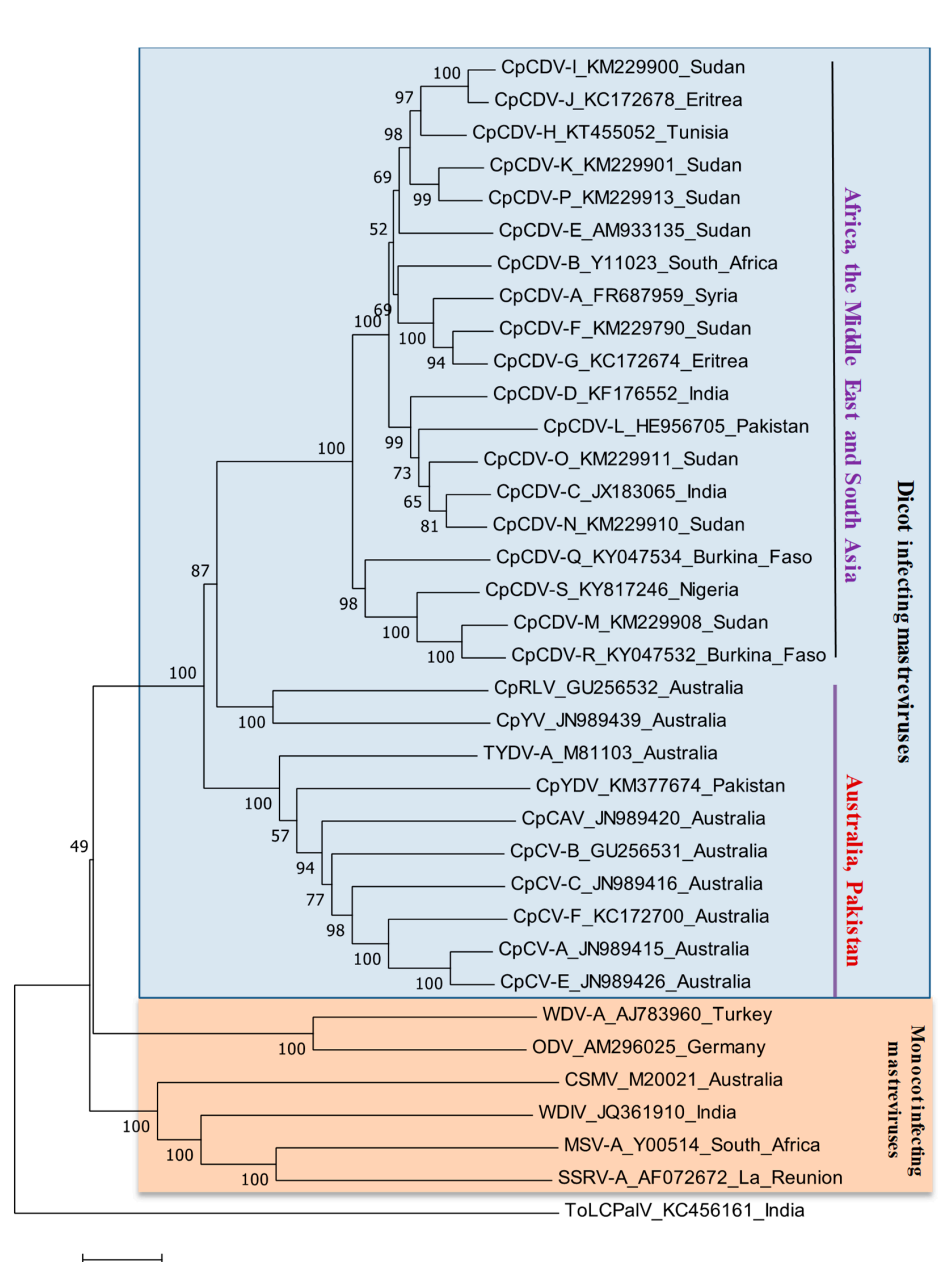
Phylogenetic relationships among infecting mastreviruses. Phylogenetic relationships among the full-length dicot infecting mastreviruses available in the GenBank. The neighbor-joining method was sued to construction of the tree with the MEGA 6 software program (http://www.megasoftware.net) and the reliability of the branches was inferred from a bootstrap analysis of 1000 replicates and only the nodes with values greater than 50% are labelled. DNA A sequence of the bipartite begomovirus species tomato leaf curl Palampur virus (ToLCPalV) was included as an outgroup.

Dicot-infecting mastreviruses are widely distributed in the chickpea-growing regions of the world, including Australia, Africa, the Middle East, and South Asia. Genetic diversity based on the whole genome visualized two major groups, one with monocot-infecting mastreviruses and the other one comprises dicot-infecting mastreviruses (Figure 4). Among the dicot-infecting mastreviruses, two clades were visualized in phylogeny analysis, with one comprising dicot-infecting viruses from Africa, the Middle East, and South Asia (CpCDV-A to CpCDV-S), and the other clade consisting of dicot-infecting mastreviruses from Australia (CpRV, CpYV, TYDV-A, CpCAV, CpCV-A, CpCV-B, CpCV-C, CpCV-E, and CpCV-F) and Pakistan (CpYDV).

Mastreviruses are also well documented for having inter/intra species recombination [57,58]. Analyses of CpCDV sequences have suggested that recombination drives the evolution of this virus. The recombination analysis performed clearly indicates the presence of (a) inter- and intra-species recombination; (b) several breaking points within the Rep, CP, and intergenic common region (ICR); and (c) clear recombination breakpoints, hot and cold spots in Rep and CP genes, respectively [34,35,45]. These frequent exchanges of genomes might have resulted in the creation of new species and strains that may evolve to threaten agriculture.

### 4.4. Biology and Interaction of Begomoviruses and Satellite Molecules with CpCDV

Mubin et al. (2012) [49] showed the first co-infection of CpCDV with two begomoviruses (cotton leaf curl Burewala virus (CLCuBuV) and tomato leaf curl Gujarat virus (ToLCGuV) and two satellites (tomato yellow leaf curl Thailand betasatellite (TYLCTHB) and potato leaf curl alphasatellite (PotLCA) in *X. strumarium*. Similarly, CpCDV was also identified with CLCuBuV in cotton plants affected by leaf curl disease [47]. Similar to begomoviruses, some mastreviruses (i.e., *Wheat dwarf India virus* (WDIV) and CpCDV) have also been found to be associated with DNA satellite molecules in the field conditions [17,18]. More interestingly, βC1 has also been shown to be a pathogenicity determinant for both begomoviruses and monocot-infecting *mastreviruses* [59,60]. More recently, the association of CpCDV-C with *Cotton leaf curl Multan betasatellite* (CLCuMB) and *Cotton leaf curl Multan alphasatellite* (CLCuMA) was observed in spinach and its ability to *trans*-replicate CLCuMB in *N. benthamiana* was demonstrated [18].

However, our attempts to *trans*-replicate tomato leaf curl new Delhi virus (ToLCNDV) DNA B and betasatellite (CLCuMuB) by CpCDV-C in *N. benthamiana* showed only typical CpCDV symptoms and no *trans*-replication (Figure 3h,i). The presence of both ToLCNDV DNA B and CLCuMuB was not detected in molecular tests performed (data not shown). Similarly, co-inoculation of WDIV with CLCuMuB showed that CLCuMuB was not maintained by WDIV in wheat [17]. Since it is well known that geminivirus-encoded Rep protein binds to iterons, which plays a key role in initiating the replication of viral DNA [31], a specific betasatellite with compatible iteron–iteron-like sequences with CpCDV is needed to understand their interactions. It is still unknown whether CpCDV can *trans*-replicate CLCuMuB or any other betasatellite. Until now, there is no information available about the frequency of these associations in CpCDV epidemics.

## 5. Virus–Vector Interactions

Members of the genus *Mastrevirus* are transmitted by leafhoppers (family Cicadellidae). The leafhopper vector of the CpCDV causing the stunt disease in India was identified as *O. albicinctus* by Horn et al. (1994) [9]. Horn et al. (1993) [11] reported that leafhopper *O. albicinctus* successfully transmitted the CpCDV to a wide range of hosts belonging to the families Solanaceae, Leguminosae, and Chenopodiaceae*,* and they found that the virus was efficiently transmitted with a median acquisition access period (AAP), inoculation access period (IAP), and latency period (LP) of 8, 2.3, and 27.7 h, respectively. By serial transmission, they also showed that the vector can transmit the virus for most of their lifespan after a two-day AAP. There was similarity between CpCDV transmission with those conditions given for MSV [61] and BCTV [62,63,64]. More recently, Akhtar et al. (2011) [65] demonstrated that CpCDV is successfully transmitted by *O. albicinctus*. Its presence was detected in inoculated chickpea plants, and the vector was confirmed by double antibody sandwich enzyme-linked immunosorbent assay (DAS-ELISA) test using specific polyclonal antibodies. Further studies on CpCDV–leafhopper transmission assays will reveal the alternative inoculum sources and CpCDV epidemics.

## 6. Detection and Diagnosis

The symptoms caused by mastreviruses in graminaceous hosts are often similar to symptoms caused by abiotic agents like nutritional deficiencies. In the case of dicot-infecting mastreviruses, it is difficult to distinguish a *mastrevirus*-infected plant from plants affected by other pathogens. In these circumstances, it is necessary to have virus-specific diagnostic reagents to detect the virus present in naturally infected plants. The diagnostics are also required to detect the virus in the insects visiting the plants in order to identity the vectors. Serological diagnostic methods such as DAS-ELISA, dot-blot ELISA, and tissue-blot immunoassay (TBIA) have been developed to detect the presence of CpCDV from infected field plant samples and viruliferous vector [10,46,65,66].

Over the past decade, rolling circle amplification (RCA) and restriction fragment length polymorphism (RFLP) has been extensively used to identify geminiviruses in most virus-infected plants. RCA was developed using the bacteriophage varphi 29 DNA polymerase, and was central in revolutionizing the detection and diagnosis of geminiviruses [67]. This technique is widely used to efficiently detect and characterize most of the dicot-infecting mastreviruses from field samples [13,14,34,35,45]. PCR-based methods involving gene-specific primers have also been developed and shown to efficiently detect CpCDV in infected plant tissues [13,14,46]. In addition, a pair of abutting primers have been utilized to amplify full-length dicot-infecting mastreviruses from Phi29 DNA polymerase-enriched DNA samples [45].

Kanakala et al. (2013) [56] detected CpCDV from field plants with a dot-blot hybridization method using a radiolabeled probe. Full-length replicative forms of CpCDV in agroinoculated plants were also detected using CpCDV-specific probes [13,14,18]. Recently, significant reductions in the costs of next-generation sequencing have accelerated use of deep sequencing for the detection and discovery of new strains of CpCDV [25,33]. Improved molecular techniques and whole-genome sequencing approaches for rapid detection of new viruses infecting chickpea offers new understanding of the evolution of CpCDV and its isolates.

All these molecular methods corroborate the importance of the extensive *Mastrevirus* diagnosis and control vector population to transmit disease. The relationships between mastreviruses infecting different host species need to be understood to develop management strategies that will prevent the further emergence of new viruses. An extensive global sampling and metagenomics analysis using next-generation sequencing of these viruses will identify the global diversity of dicot-infecting mastreviruses and inform better strategies for diagnostics and disease management.

## 7. Management Strategies

### 7.1. Host Plant Resistance

In the past decade, an upsurge of chickpea viral diseases has been experienced, resulting in economic losses of chickpea production across the growing regions. Successful plant breeding programs for disease resistance depend on the successful identification of sources of resistance and the incorporation of resistance genes into commercial varieties [68,69]. Chickpea stunt disease is widespread in the old world, and causes considerable yield loss. The disease has been recognized as a serious challenge to chickpea cultivation, and resistance-breeding programs are being taken up. However, they are dependent on natural occurrences of the disease, as evaluation by inoculation through the vector is often cumbersome. At present, evaluation of CpCDV resistance is conducted on the basis of field screening of chickpea germplasm.

Among 10,000 germplasm lines screened for resistance to stunt disease, two lines (GG669 and ICCC10) were found to be field-resistant to CpCDV [12]. More recently, Kanakala et al. (2013) [56] has developed an agroinoculation technique to screen chickpea genotypes against CpCDV. This technique involves the construction of a complete tandem repeat CpCDV construct and the delivery of full-length CpCDV into germinated chickpea seed through *Agrobacterium tumefaciens*. Over 70 genotypes screened genotype SCGP-WR-29, which showed resistance in the field condition but exhibited 80% incidence under agroinoculation. Three agroinoculated genotypes (L-550, GNG-1499 (Gauri), and IPC 09-07) showed virus resistance and did not express any symptoms, and plants remained alive compared to susceptible genotypes. More interestingly, resistant plants were shown to be virus-free under PCR tests. These kinds of resistance screening tests have yet to be adopted to generate CpCDV-resistant cultivars on a wide scale. In the same study, an objective scoring to assess the response of chickpea genotypes to CSD by agroinoculation of CpCDV construct was also developed.

### 7.2. Genetic Engineering Approaches

RNA interference (RNAi) is a very promising strategy that has been employed to control both plant viruses and insect vectors [70,71]. Hairpin RNAi constructs containing sequences of CpCDV Rep and MP genes were stably expressed in *N. benthamiana* to provide immunity to CpCDV inoculation [72]. Baltes et al. (2015) [73] demonstrated a novel strategy for engineering resistance to BeYDV/CpCDV-B with a clustered regularly interspaced short palindromic repeats/CRISPR-associated (CRISPR-Cas) prokaryotic immune system. Transgenic *N. benthamiana* plants expressing CRISPR-Cas reagents and challenged with BeYDV had reduced virus load and symptoms [73]. However, until now, there have been no reports on the transgenic control of any dicot-infecting *Mastrevirus* in chickpea. This will require significant progress in tissue culture and transformation technologies to CpCDV-resistant chickpea genotypes.

Similarly, RNAi has been successfully demonstrated in other leafhopper insect vectors. Silencing/knockdown of insect genes *laccase-2/peptidoglycan recognition protein* (*PGRP-LC*) resulted in significant mortality in leafhoppers [74,75]. Until now, there have been no RNAi-based silencing experiments studied in *Mastrevirus* insect vector. The identification of such candidate genes and the development of transgenic plants expressing dsRNA/SiRNA that target insect genes are necessary to control insect virus transmission under field conditions. Recent genome editing tools like CRISPR/Cas9 are highly suggested to modify virus/vector genes in order to develop effective resistance against CpCDV/*O. albicinctus*.

## 8. Future Prospects

CpCDV continues to be a threat to chickpea production worldwide. Although the virus was first reported in India in the year 1993, today CpCDV has been reported in Africa, the Middle East, and Australia, because of the polyphagous and widespread insect vector. At present, mixed infections, the emergence of new strains, and inter/intra recombinations among CpCDV strains/species might have increased its host range and caused new epidemics. Some major questions remain to be answered concerning (1) Mastrevirus–satellite interactions, (2) virus–host–insect interactions, (3) insect vector and its endosymbiont’s efficacy in virus transmission, and (4) the discovery of CSD-resistant chickpea varieties. Considering the continuing new reports of CpCDV strains from new hosts and regions of the world, and given the importance of the fourth most widely grown pulse, continued research is needed to understand the biology, ecology, and epidemiology of CpCDV and its insect vector.

Over the past few years, a very low number of resistant chickpea varieties were screened through virus inoculations [12,56]. One possible new strategy that we can consider for engineering resistance against chickpea-infecting geminiviruses is genome editing through CRISPR/Cas9. Some recent studies exploited CRISPR/Cas9 technology, and could impart molecular immunity in single/mixed geminivirus infections by targeting the most conserved nonanucleotide sequence (TAATATTAC) present in the LIR or coding regions of the viral genome [73,76,77,78]. Finally, pathogen-derived resistance strategies or gene editing methods need to be utilized to facilitate the development of chickpea cultivars with resistance to chickpea stunt disease.

## Figures and Tables

**Table 1:** Geographical distribution and host range of dicot infecting mastreviruses.

Dicot Infecting Mastreviruses	Countries	Host Plant Species	Reference
**CpCV**			
**CpCV-A**	Australia	*C. arietinum*	[35,44]
**CpCV-B**	Australia	*C. arietinum*	[35,45]
**CpCV-C**	Australia	*C. arietinum*	[45]
**CpCV-E**	Australia	*C. arietinum, P. vulgaris*	[35,45]
**CpCV-F**	Australia	*C. arietinum*	[35]
**CpCAV**	Australia	*C. arietinum*, *P. vulgaris*	[45]
**CpCDV**			
CpCDV-A	Syria, Iran, Turkey, Tunisia, Egypt	*C. arietinum,C. lanatus*, Squash, *P. sativum*	[23,24,35,44,45]
CpCDV-B	Pakistan, South Africa	*P. vulgaris, C. arietinum*	[13,21]
CpCDV-C	India, Sudan, Pakistan	*C. arietinum*, cucumber, *G. hirsutum*, *G. arboretum,* Okra, *C. annuum*, *V. faba*, *L. culinaris, S. lycopersicum*, Spinach (*S. oleracea*)	[11,13,14,18,22,34,35,50,51,52]
CpCDV-D	India, Pakistan, Sudan, Morocco	*C. arietinum*, *P. sativum,L. culinaris*	[35]
CpCDV-E	Sudan	*C. arietinum*, *V. faba*	[34]
CpCDV-F	Sudan, Pakistan, Syria, Yemen, Oman, Eritrea	*C. arietinum*, Pepper, *L. culinaris*, *V. faba*	[34,35,48]
CpCDV-G	Eritrea	*C. arietinum*	[35]
CpCDV-H	Sudan, Pakistan, Eritrea, Tunisia	*C. arietinum*, *P. sativum*, *L. culinaris*, *V. faba*	[34]
CpCDV-I	Sudan, Eritrea	*C. arietinum*	[34]
CpCDV-J	Eritrea	*C. arietinum*	[34]
CpCDV-K	Sudan, Eritrea	*C. arietinum*	[34]
CpCDV-L	Pakistan	*G. hirsutum, G. arboreum*	[47]
CpCDV-M	Sudan	*C. arietinum*	[34]
CpCDV-N	Sudan	*C. arietinum*	[34]
CpCDV-O	Sudan	*C. arietinum*	[34]
CpCDV-P	Sudan	*C. arietinum*	[34]
CpCDV-Q	Burkina Faso	*C. papaya*	[25]
CpCDV-R	Burkina Faso	*S. lycopersicum*	[25]
CpCDV-S	Nigeria	*C. papaya*	[33]
**CpYDV**	Pakistan	*C. arietinum*	[36]
**CpRLV**	Australia	*C. arietinum*	[44]
**CpYV**	Australia	*C. arietinum*	[45]
**TYDV**	Australia	Tobacco, Wild radish, *P. vulgaris*, *C. arietinum,* Turnip weed (*R. rugosum*)	[35,38,44]
